# Effects of social stories intervention for children and adolescents with autism spectrum disorders: A protocol for a systematic review and meta-analysis of randomized controlled trials

**DOI:** 10.1097/MD.0000000000022018

**Published:** 2020-09-11

**Authors:** Tingting Chen, Wenxu Yang, Qiu Wang, Ying Zhang, Zhigui Ma

**Affiliations:** a Key Laboratory of Birth Defects and Related Diseases of Women and Children (Sichuan University), Ministry of Education, West China Second University Hospital, Sichuan University, Chengdu, Sichuan; b Department of Pediatric Cardiology; c Department of Child Health Care, Chengdu Women's and Children's Central Hospital, School of Medicine, University of Electronic Science and Technology of China; d Department of Pediatric Rehabilitation, West China Second University Hospital, Sichuan University; e Department of Pediatric Respirology, Chengdu Women's and Children's Central Hospital, School of Medicine, University of Electronic Science and Technology of China, Chengdu, China.

**Keywords:** adolescent, autism spectrum disorders, children, meta-analysis, social stories, systematic review

## Abstract

**Background:**

Autism spectrum disorder (ASD) is a common neurodevelopmental disorder, which lacks specific medical treatment. Intervention is the key point of rehabilitation training for ASD. Social stories (SS) are a commonly used intervention practice in individuals with ASD. However, there is mixed evidence on the effectiveness of SS. Thus, the objective of this systematic review and meta-analysis is to assess studies of the effects of SS for children and adolescents with ASD.

**Methods:**

To identify relevant studies, we will search PubMed, EMBASE, Cochrane Library, Web of Science, Google Scholar and trials registers (the World Health Organization International Clinical Trial Registration Platform, ClinicalTrials.gov, and Chinese Clinical Trial Register) from inception to May 2020. In addition, we will also perform handsearching of grey literature, such as conference proceedings and academic degree dissertations. Only the randomized control trials will be accepted, no matter what the languages they were reported. We will first focus on the effectiveness of the intervention on the behavior of the targets. Then we will do further analysis of the study design, including the length and intensity of intervention, the characteristics of participants and interveners, the methods of assessment, the place, the medium, and the economic feasibility. Two independent reviewers will carry out literature identification, data collection, and study quality assessment. Discrepancies will be resolved by a third reviewer. The Cochrane Risk of Bias Tool will be used to evaluate the risk of bias of the randomized controlled trials. Data analysis will be calculated using the STATA 13.0 software.

**Result:**

This study will offer new evidence whether the SS is an appropriate intervention of benefiting the children and adolescents with ASD, and to determine which factors affect the effectiveness of SS.

**Conclusion:**

The conclusion drawn from this systematic review will benefit the children and adolescents with ASD.

## 1 Introduction

Autism spectrum disorder (ASD) refers to a heterogeneous neurodevelopmental condition characterized by impairment in reciprocal social interaction and communication, together with repetitive and restrictive behaviors.^[1]^ There are wide clinical characteristics with ASD, including variable degrees of communication skills, motor abnormalities, intellectual impairments, and various comorbidities.^[2–4]^ Social impairments are common symptoms in ASD, which encompass diverse impaired social cognitive processes, such as dullness of orienting reflex, incomprehension of others’ cognitive states or actions and inappropriate self-referential thought.^[5]^ As the prevalence of ASD continues to rise, ^[4,6]^ it reaches 1:54 children in the United States. ^[7]^ The increased risk for somatic and psychiatric illness, reduced quality of life and premature mortality suggest that ASD affects many families and represents a serious public health problem.

The appropriate intensive behavioral therapies are effective in reducing disability in many children with ASD. Developed in 1993,^[8]^ Social stories (SS) describes various social situation consisting of individualized phrases or stories that specify how a person should act in certain contexts or situations, which is accurate, convincing and easy to understand by children with ASD. The objective is to assist children with ASD in teaching socially appropriate behaviors, and reduce disruptive behaviors.^[9,10]^ Due to flexibility and capacity for individualization, they tend to have high acceptance and applicability.^[10]^

There is mixed evidence on the effectiveness of SS. Previous literatures suggested that SS can improve understanding and performance in social situations.^[11,12]^ Other researchers had come to inconsistent conclusions, noting uncertainty about the efficacy of SS for children with ASD.^[13,14]^ A number of systematic reviews have been conducted on the effect of SS intervention for children with ASD.^[13–15]^ However, there is currently no systematic review and meta-analysis of randomized controlled trials (RCTs). The primary aim of this study is to undertake a comprehensive systematic review and meta-analysis to evaluate SS in children with strong experimental designs, and tease out what factors influence their effectiveness.

## 2 Methods

### 2.1 Design and registration

This systematic review is registered on the international prospective register of systematic reviews (PROSPERO). Registration number is CRD42020189708. This protocol is conducted according to the Preferred Reporting Items for Systematic Reviews and Meta-Analysis Protocol (PRISMA-P) statement guidelines^[16]^ and the Cochrane Handbook for Systematic Reviews of Interventions^[17]^.

### 2.2 Criteria for including studies in this review

#### 2.2.1 Types of studies

To evaluate the efficacy of SS in the intervention of ASD, this paper only reviewed the RCT between SS and the control group, without any restrictions on blinding, language, date of transmission or type of publication. Non-RCTs, quasi-RCTs, retrospective studies, case reports, non-controlled trials, and animal mechanism studies will be excluded.

#### 2.2.2 Types of participants

Diagnosis of ASD patients (<18 years of age) will be included in the analysis, regardless of their gender, ethnicity, and background, according to the International Classification of Diseases (ICD) 11,^[18]^ or the Diagnostic and Statistical Manual of Mental Disorders (DSM)-V,^[19]^ research diagnostic criteria.

#### 2.2.3 Types of interventions

We define SS as the experimental intervention. SS included in this study will follow the Gray's guidelines^[8,20]^ as the primary intervention. If the studies included use more than one intervention, the data of SS should be separated.

There will be no restrictions with respect to the type of comparator. The comparisons are likely to include: no intervention, sham intervention, other active procedures and SS in addition to active treatment compared with the same active treatment.

#### 2.2.4 Types of outcome measures

The primary outcome will be the effect of improvements in target behaviors, which include 3 broad categories: restricted and repetitive behaviors, impairment in social communication, and appropriate life skills.

The secondary outcomes will consider study design, the length and intensity of intervention, the characteristics of participants and interveners, the use of assessment, place, medium and economic feasibility.

### 2.3 Data sources and search strategy

We will search electronic databases including PubMed, EMBASE, Cochrane Library, Web of Science, Google Scholar and trials registers (the World Health Organization International Clinical Trial Registration Platform, ClinicalTrials.gov and Chinese Clinical Trial Register) from inception to May 2020. Grey literature, such as Conference proceedings and academic degree dissertations will be manually searched. For a comprehensive search, a search strategy that combines MeSH terms and free words will be adopted. Search strategy in PubMed is shown in Table 1.

**Table 1:**
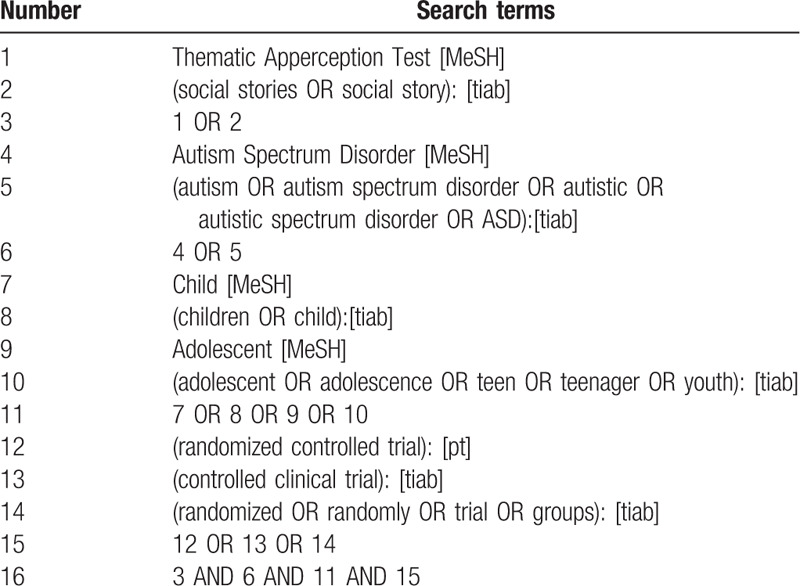
Search strategy for PubMed.

### 2.4 Data collection and analysis

#### 2.4.1 study selection

**Figure 1:**
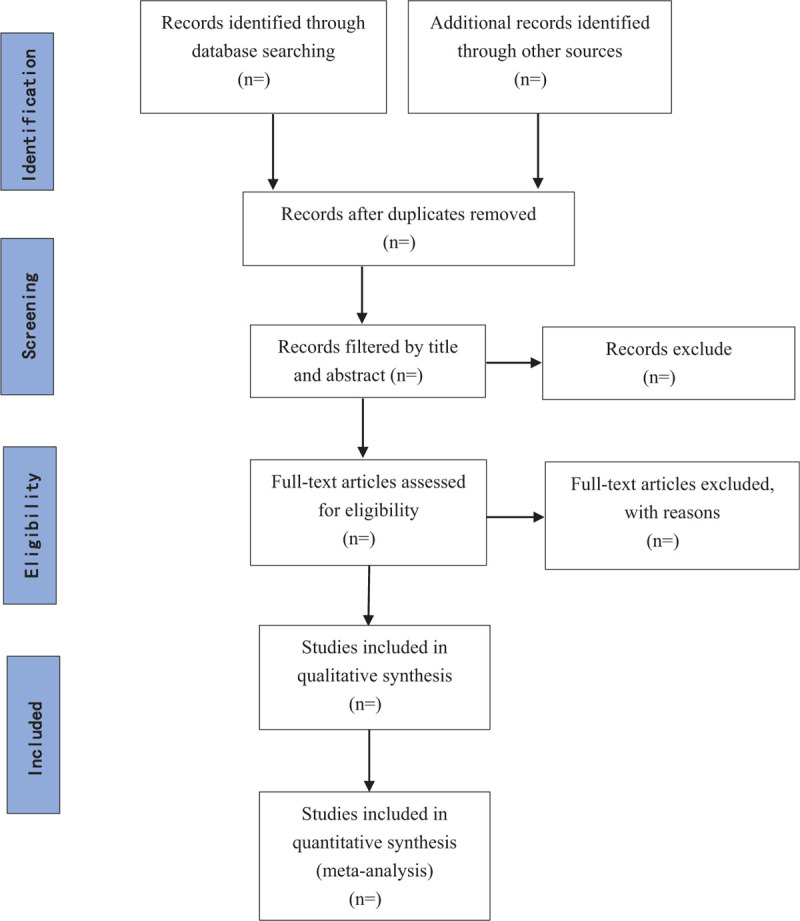
Flow chart of study flow from systematic search to selection process.

In the literature screening process, we will use EndNote X7 software. Two reviewers will independently assess all relevant studies and select eligible articles that meet inclusion criteria based on the title and abstract. The full texts of articles which are potentially eligible will be examined for further evaluation. In case of a discrepancy between the 2 review authors, it will be discussed and made an agreement with the third author. A flow diagram for the selection process will be developed using the Preferred Reporting Items for Systematic Reviews and Meta-Analysis guidelines. (Fig. 1).^[21]^

#### 2.4.2 Data extraction

Two authors will extract the data needed and fill out the data extraction form independently. The data form will include first author, year of publication, region, sex, age, number of cases, number of controls, intervention details, completion of intervention, missing participants, intervention time, control, outcome, and conflicts of interest. When encountering a necessary outcome that is inaccessible for direct data extraction, we will attempt to obtain information from the corresponding authors by e-mail. Any disagreements will be discussed and resolved in discussion with a third author.

#### 2.4.3 Assessment of risk of bias

Following the guidance in the latest version of Cochrane Handbook for systematic reviews of interventions,^[22]^ two reviewers will independently assess the risk of bias for each included study with version 2 of the Cochrane risk-of bias tool for randomized trials, ROB 2.^[23]^ We will examine five domains, including bias from the randomization process, bias due to deviations from intended interventions, bias due to missing outcome data, bias in measurement of the outcome, and bias in selection of the reported result. If there is insufficient detail to assess the risk of bias, we will contact study authors by e-mail. The third reviewer will arbitrate in the case of any disagreement.

#### 2.4.4 Data synthesis and statistical analysis

In accordance with Higgins et al,^[24]^ only the first phase of the data will be included in the random crossover trial. If the primary result has missing or incomplete data, we will contact the author to obtain the missing data. Statistical analyses will be performed using STATA 13.0 software. The risk ratio with 95% confidence interval will be used to assess dichotomous data. Continuous outcomes will be expressed as standardized mean difference along with its 95% confidence interval. We will synthetize primary studies to explore heterogeneity descriptively rather than statistically. The statistical heterogeneity among studies was assessed with the *Q*-test and *I*^2^ statistics. If significant heterogeneity is found, the random-effects model will be used to estimate the data, otherwise, the fixed-effects model will be used. Funnel plot and Egger test will be used to detect the potential reporting biases if at least 10 studies are included.

#### 2.4.5 Sensitivity analysis and subgroup analysis

To check the robustness of pooled outcome results, sensitivity analyses will be conducted to assess how including and excluding studies influences the results, when studies are adequate. We will repeat the analysis after excluding cross-over trials and trials with a high risk of bias. If appropriate data are available, subgroup analyses will be exploratory based on geographical location, age, control interventions and different outcomes.

## 3 Discussion

ASD symptoms often accompany the whole life of individuals. Without special intervention and rehabilitation, most of them will have lifelong intellectual and mental disability, which will have a great impact on their social function.^[25,26]^ SS could be a promising intervention to improve children's appropriate behavior and social skills and reduce destructive behavior. However, there is an ongoing debate regarding the effectiveness of SS in children with ASD due to uncertainty of factors affecting benefits. As we know, the current study will be the first meta-analysis of RCT for efficacy of SS in children with ASD. We will comprehensively evaluate randomized data characteristics, including detailed information of interventions and primary and secondary outcomes. According to the Cochrane method, this study is based on the analysis of RCT evidence, searching and screening the main electronic literature database, providing therapists with more convincing evidence in decision-making, to better guide intervention.

## Author contributions

**Conceptualization:** Tingting Chen, Zhigui Ma

**Data curation:** Tingting Chen, Wenxu Yang, Zhigui Ma

**Formal analysis:** Qiu Wang, Ying Zhang

**Funding acquisition:** Tingting Chen

**Methodology:** Tingting Chen, Wenxu Yang,Qiu Wang

**Software:** Tingting Chen, Ying Zhang

**Supervision:** Zhigui Ma

**Writing – original draft:** Tingting Chen, Wenxu Yang

**Writing – review & editing:** Tingting Chen, Zhigui Ma
